# Relationship between prognostic nutritional index and asthma: a cross-sectional analysis

**DOI:** 10.3389/fnut.2025.1467270

**Published:** 2025-02-06

**Authors:** Zhimeng Jiang, Xingyu Zhu, Huixin Jiang, Donglin Zhao, Jianwei Tian

**Affiliations:** ^1^Graduate School of Hebei North University, Zhangjiakou, Hebei, China; ^2^Department of Gastroenterology, Air Force Medical Center, Chinese People’s Liberation Army, Beijing, China; ^3^Department of Cardiovascular Medicine, Air Force Medical Center, Chinese People’s Liberation Army, Beijing, China; ^4^Haiyuan College of Kunming Medical University, Kunming, Yunnan, China

**Keywords:** prognostic nutritional index, asthma, nutritional status, inflammatory, cross-sectional study, National Health and Nutrition Examination Survey

## Abstract

**Background and objective:**

Asthma is a chronic disease characterized by inflammation of the airways. The association between nutritional status, inflammation, and asthma has been well-documented, yet the relationship between the Prognostic Nutritional Index (PNI) and asthma remains unclear. This is a study to see whether there is a relationship between PNI and asthma prevalence.

**Methods:**

The present study employed data from the National Health and Nutrition Examination Survey (NHANES) between 2017 and 2020, including a total of 7,869 adult participants were included in the analysis. Participants were categorized into four quartiles based on PNI levels. A multivariable regression model was employed for the purpose of evaluating the correlation between PNI and asthma. In order to ascertain the stability of the association across different populations, subgroup analyses were performed.

**Results:**

Higher PNI levels were significantly associated with lower asthma prevalence. In the complete adjusted model, each additional unit of PNI was associated with a 3% reduction in the prevalence of asthma [0.97 (0.95, 0.99)]. Trend analysis indicated a significant negative correlation between PNI and asthma (*p* for trend = 0.0041). Subgroup analyses showed a consistent negative association across different populations.

**Conclusion:**

The findings of our study indicated that lower PNI values were linked to an elevated odds prevalence of asthma. Early nutritional intervention and inflammation management in high-risk populations with low PNI may reduce the incidence and severity of asthma. Future prospective studies are needed to confirm this relationship.

## 1 Introduction

Asthma is a common chronic inflammatory airway disease that can cause bronchospasm, leading to breathing difficulties and, in severe cases, can be life-threatening (1). It is reported that asthma has the highest prevalence among chronic respiratory diseases, with a global prevalence reaching 262.4 million and rising (2). The 2017 National Health Interview Survey in the United States indicated that asthma was responsible for 1.6 million hospital admissions and 183,000 instances of emergency care among individuals with chronic airway inflammatory diseases (3). Asthma cannot be completely cured, and its persistent chronic airway inflammation and hyperresponsiveness (4), often lead to poor symptom control, frequent hospitalizations, and significant economic burdens (5). Previous studies have indicated that poor asthma control and frequent exacerbations increase the risk of cardiovascular diseases in patients with asthma (6). Moreover, respiratory infections also can further impair lung function and exacerbate asthma symptoms (7). Additionally, it is a well-documented fact that individuals suffering from asthma frequently have associated metabolic diseases, including insulin resistance and obesity. These metabolic problems have been shown to exacerbate asthma symptoms and result in suboptimal treatment outcomes (8, 9). Lastly, individuals afflicted with asthma, particularly those experiencing inadequate symptom management, are predisposed to mental health complications, including anxiety and depression. These psychological ailments can impede the efficacy of asthma treatment regimens (10, 11). This underscores the significance of effective asthma management.

The Prognostic Nutritional Index (PNI), calculated from the product of serum albumin and lymphocyte count, provides a comprehensive assessment of an individual’s nutritional, immune, and inflammatory status. Existing studies indicate that PNI has good predictive value in evaluating cancer-related prognosis (12–15). PNI can also be used for risk assessment in various diseases, such as cognitive function in the elderly (16), complications and mortality in stroke patients (17), prognosis in chronic renal failure patients (18), and mortality in coronary artery disease (19), showing broad potential applications.

Prior studies have indicated that nutritional status, inflammatory response, and asthma are closely related (20, 21). Malnutrition, often accompanied by hypoalbuminemia, weakens the immune system, making it difficult for the body to effectively resist and regulate inflammation (22). A number of nutrients have been demonstrated to exert a significant influence on the onset and development of obstructive pulmonary diseases (23, 24). Since asthma is a chronic inflammatory disease, assessing nutritional status and inflammation is essential for managing the condition. PNI, as an integrated measure of inflammation and immune system status, can provide insights into the systemic inflammatory burden in asthma patients, potentially identifying those at risk for poor control or exacerbations. At this time, the relationship, if any, between PNI and asthma remains unclear. Accordingly, the present study employed data from adult participants in the National Health and Nutrition Examination Survey (NHANES) to investigate the relationship between PNI and asthma.

## 2 Methods

### 2.1 Study population

NHANES is a nationally representative data set on the nutrition and health status of non-institutionalized US residents. NHANES employs multistage probability sampling methodology to provide a representative sample of the US population for the purpose of monitoring and evaluating the nation’s health status in relation to nutrition. The NHANES study is approved by the National Center for Health Statistics (NCHS) with strict ethical review, ensuring that all participants signed written informed consent.

**Figure 1:**
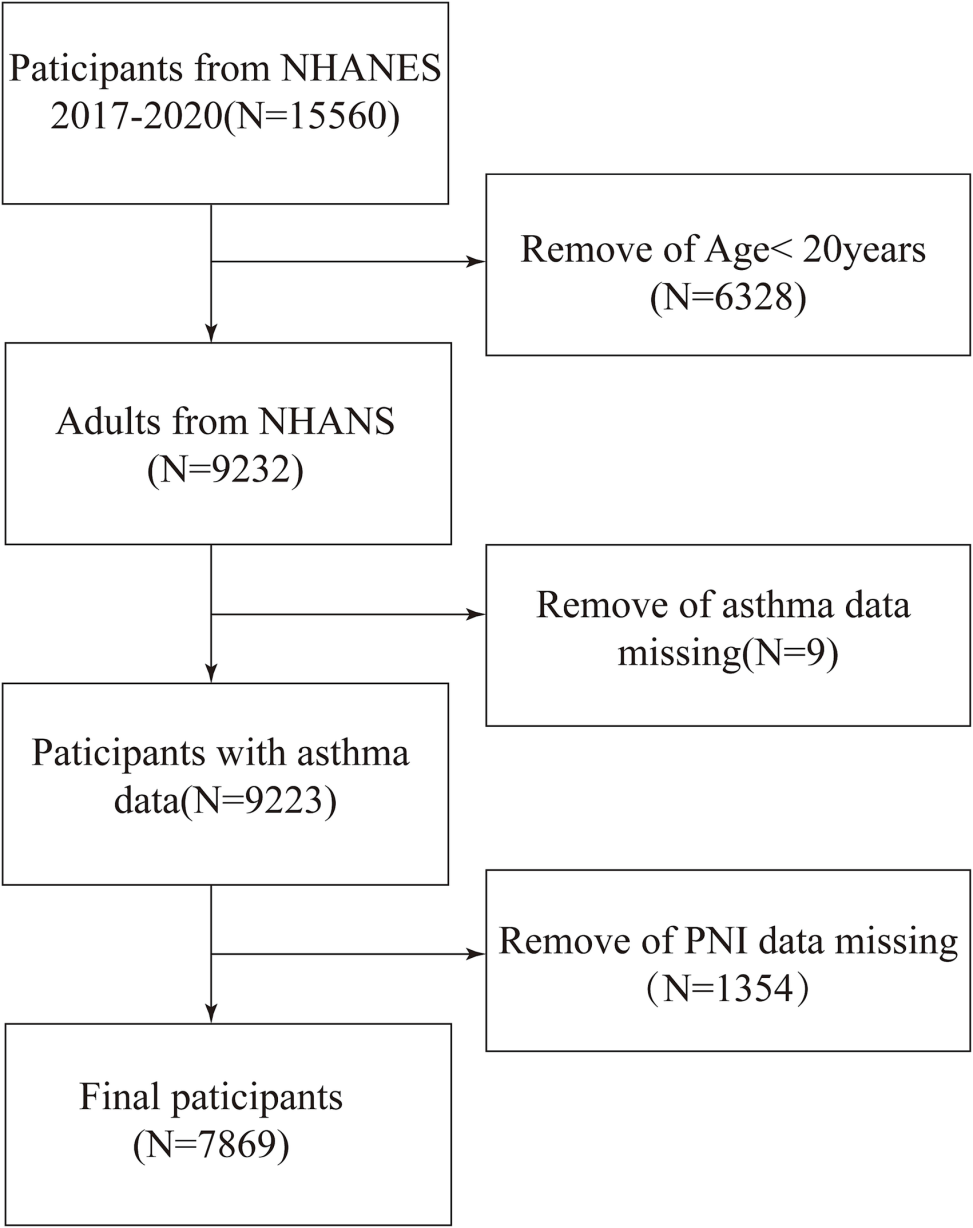
Flow diagram indicating study population. NHANES, National Health and Nutrition Examination Survey. PNI, Prognostic Nutritional Index.

Our study used NHANES data from 2017 to 2020. Initially, we recruited 15,560 eligible participants. We removed 6,328 participants below the age of 20 years, 9 participants with missing asthma information, and 1,354 participants with missing PNI data, ultimately including 7,869 participants who met the criteria (Figure 1).

### 2.2 Exposure variables and outcome definition

The exposure factor in this study was PNI, calculated using the formula: PNI = [10 × albumin (g/dL)] + [0.005 × absolute lymphocyte count (10^3^ cells/μL)] (25). Participants were analyzed by categorizing PNI into quartiles. Albumin concentration was measured using bromocresol purple dye, and lymphocyte count was measured using a Beckman Coulter DxH 800 instrument. The outcome variable was the occurrence of asthma, assessed through self-reported questionnaires asking, “Have you ever been told you have asthma?” If participants answered “yes, “they were determined to have asthma.

### 2.3 Covariates

We considered multiple potential covariates that could confound the relationship between PNI and asthma. The covariates included demographic variables (gender, age, race, education level, poverty-income ratio (PIR), marital status), lifestyle factors (alcohol consumption, smoking status, moderate work activity level), clinical measurements (white blood cell count, platelet count, total cholesterol level, high-density lipoprotein cholesterol level, low-density lipoprotein cholesterol level, triglyceride level, body mass index (BMI), serum calcium, systolic blood pressure, diastolic blood pressure, fasting blood glucose, glycated hemoglobin), and the presence of diabetes, hypertension, and arthritis. All NHANES data used in the study are publicly available at https://www.cdc.gov/nchs/nhanes/.

### 2.4 Statistical analysis

All statistical analyses were conducted following the guidelines of the Centers for Disease Control and Prevention (CDC), using appropriate NHANES sample weights. For continuous variables, variance-weighted analysis was used; for categorical variables, weighted chi-square tests were used to evaluate differences between PNI quartiles. To assess the association between PNI and asthma, multivariable regression models were employed. In Model 1, no covariates were included in the adjustments. Model 2 included adjustments for the covariates of age, gender, and race. In Model 3, all of the covariates previously mentioned were included as adjustment variables including demographic variables, lifestyle factors, and clinical measurements. Sensitivity analysis was conducted by grouping PNI into quartile levels. Smooth fitting curves evaluated the nonlinear relationship between PNI and asthma. If a nonlinear relationship was observed, a two-log likelihood ratio test was employed to ascertain the threshold effect size and to test for significant threshold effects. Lastly, stratified multivariable logistic regression models were used for subgroup analysis by gender, age, race, BMI, hypertension, and diabetes to assess the stability of the association between PNI and asthma. A *p*-value <0.05 was considered significant. All analyses were conducted using R version 4.1.3 and Empower software.

## 3 Results

### 3.1 Baseline characteristics

Table 1 presented the weighted distribution of all clinical characteristics of the included participants, stratified by PNI quartiles. A total of 7,869 adult participants were enrolled in the study, the mean age of which was 48.43 ± 17.31 years, 48.33% of whom were male, and 51.67% female. The PNI quartile ranges were 21–38, 38–41, 41–43, and 43–54. The overall prevalence of asthma was 15.42%, with quartiles 1, 2, 3, and 4 having prevalence rates of 18.30, 15.07, 14.98, and 13.53%, respectively.

**Table 1:** Baseline characteristics of the study population.

Quartiles of prognostic nutritional index					
Characteristic	Q1 (21–38) *N* = 1965	Q2 (38–41) *N* = 1969	Q3 (41–43) *N* = 1967	Q4 (43–54) *N* = 1968	*p*-value
Age (years)	52.05 ± 17.15	51.74 ± 17.09	48.75 ± 16.88	43.07 ± 16.60	<0.0001
Ratio of family income to poverty	2.86 ± 1.58	3.05 ± 1.56	3.11 ± 1.51	3.24 ± 1.55	<0.0001
White blood cell count (10^3^cells/μL)	7.54 ± 2.35	7.34 ± 2.90	7.18 ± 7.20	7.29 ± 2.03	0.0782
Platelet count (10^3^cells/μL)	259.61 ± 72.84	247.28 ± 64.14	242.26 ± 58.11	240.66 ± 57.43	<0.0001
Hs-CRP (mg/L)	7.32 ± 12.73	4.01 ± 7.58	2.86 ± 4.05	2.11 ± 3.10	<0.0001
Total calcium (mg/dL)	9.05 ± 0.35	9.21 ± 0.32	9.33 ± 0.32	9.48 ± 0.31	<0.0001
Cholesterol (mg/dL)	181.88 ± 41.03	187.96 ± 40.60	188.52 ± 40.44	189.30 ± 40.82	<0.0001
Triglycerides (mg/dL)	139.85 ± 113.00	138.83 ± 89.68	139.55 ± 96.87	141.04 ± 106.12	0.9117
HDL-Cholesterol (mg/dL)	53.11 ± 15.36	54.11 ± 16.51	53.77 ± 15.58	54.01 ± 15.86	0.2440
LDL-Cholesterol (mg/dL)	103.86 ± 36.13	109.11 ± 36.34	109.90 ± 36.06	110.17 ± 35.60	<0.0001
Glycohemoglobin (%)	5.95 ± 1.23	5.75 ± 1.01	5.64 ± 0.84	5.49 ± 0.71	<0.0001
Fasting glucose (mg/dL)	115.44 ± 40.68	110.75 ± 31.32	108.39 ± 25.03	105.08 ± 21.56	<0.0001
Systolic pressure (mmHg)	122.85 ± 19.60	122.56 ± 17.59	121.76 ± 16.50	121.49 ± 15.72	0.0421
Diastolic pressure (mmHg)	74.56 ± 11.71	74.47 ± 10.70	74.29 ± 10.21	74.02 ± 10.44	0.3931
Body mass index (kg/m^2^)	25.89 ± 8.14	25.67 ± 7.83	26.28 ± 8.26	25.98 ± 8.28	0.1318
Gender (%)					<0.0001
Male	30.06	41.48	50.87	64.09	
Female	69.94	58.52	49.13	35.91	
Race (%)					<0.0001
Mexican American	9.52	7.65	8.54	8.52	
Other Hispanic	8.20	7.03	7.96	7.59	
Non-Hispanic White	55.98	64.46	63.69	66.78	
Non-Hispanic Black	17.43	12.04	9.03	6.61	
Other race	8.86	8.82	10.78	10.50	
Education level (%)					<0.0001
Less than high school	13.83	10.79	11.45	8.89	
High school	29.10	28.40	24.28	26.35	
More than high school	57.06	60.81	64.27	64.76	
Marriage (%)					0.2209
Yes	60.86	61.12	63.82	62.41	
No	39.14	38.88	36.18	37.59	
Smoking (%)					0.0056
Yes	43.88	39.32	44.09	43.85	
No	56.12	60.68	55.91	56.15	
Drink (%)					0.2101
Yes	12.63	12.10	11.68	13.69	
No	87.37	87.90	88.32	86.31	
Moderate work activity (%)					<0.0001
Yes	43.36	48.31	49.90	51.85	
No	56.64	51.69	50.10	48.15	
High blood pressure (%)					<0.0001
Yes	40.89	33.90	33.39	25.44	
No	59.11	66.10	66.61	74.56	
Diabetes (%)					<0.0001
Yes	18.16	12.92	10.72	6.87	
No	81.84	87.08	89.28	93.13	
Arthritis (%)					<0.0001
Yes	35.77	30.00	29.47	20.24	
No	64.23	70.00	70.53	79.76	
Asthma (%)					0.0006
Yes	18.30	15.07	14.98	13.53	
No	81.70	84.93	85.02	86.47	

Mean ± SD for continuous variables: the *p* value was calculated by the weighted linear regression model; (%) for categorical variables: the p value was calculated by the weighted chi-square test. HDL-Cholesterol, high-density lipoprotein cholesterol; LDL-Cholesterol, low-density lipoprotein cholesterol; Hs-CRP, High-Sensitivity C-Reactive Protein.

### 3.2 Association between PNI and asthma

**Figure 2:**
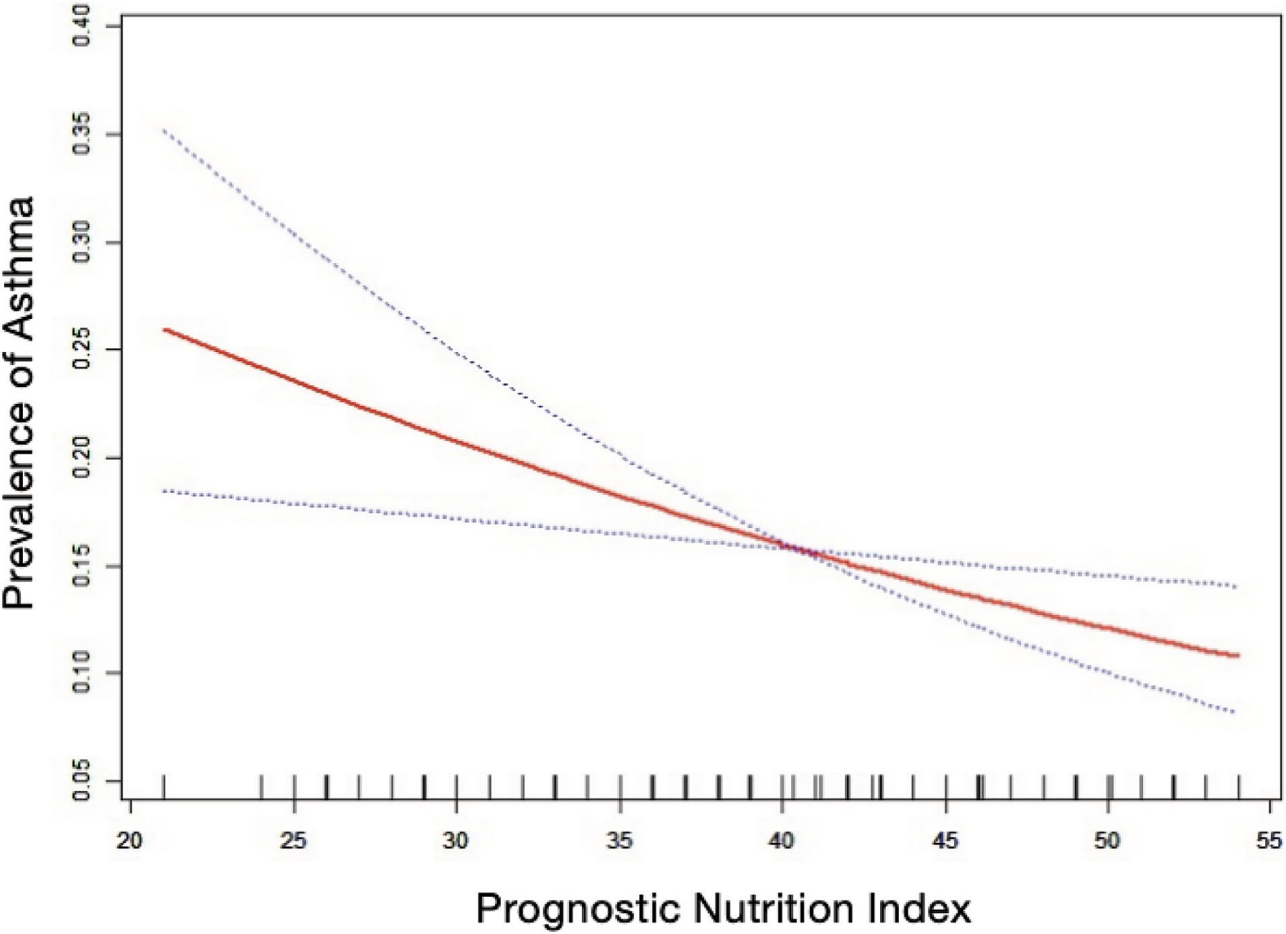
The nonlinear associations between prognostic nutritional index and asthma. The solid red line illustrates the smooth curve fit between the variables. The blue bands represent the 95% confidence interval derived from the fit.

**Figure 3:**
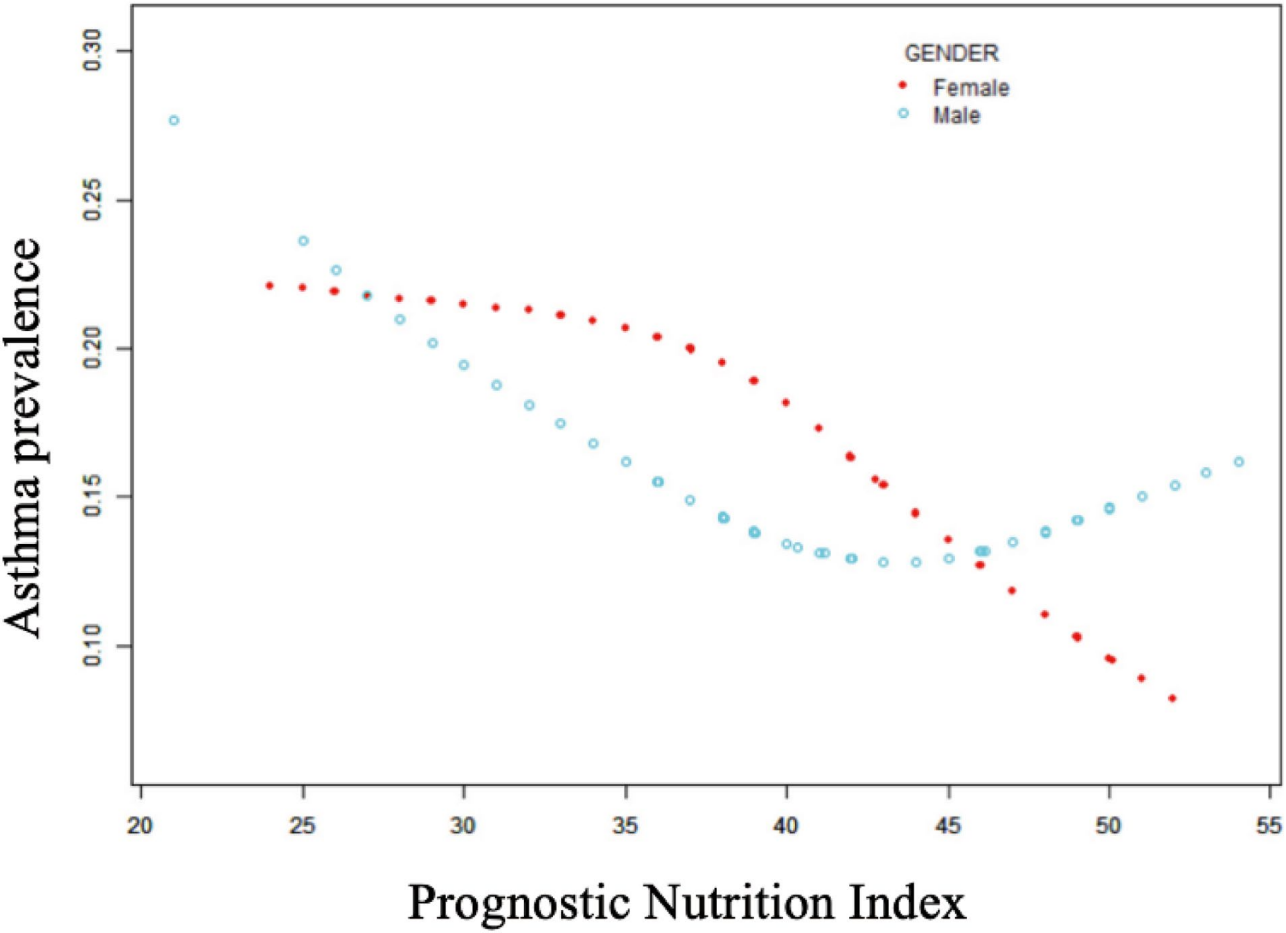
Non-linear relationship between prognostic nutritional index and asthma in different age subgroups.

**Figure 4:**
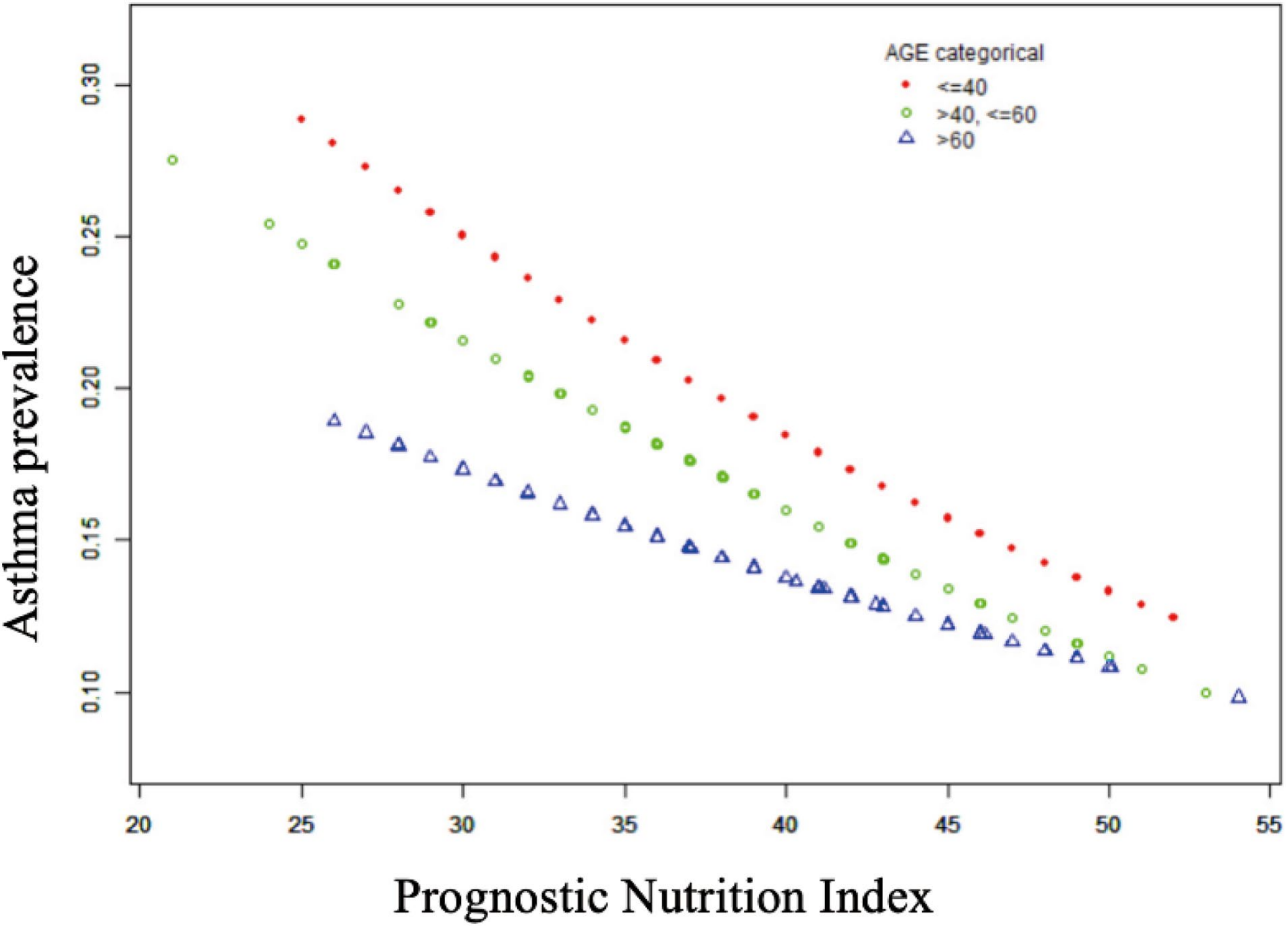
Non-linear relationship between prognostic nutritional indices and asthma across gender subgroups.

Table 2 shows that elevated PNI values were observed to be inversely associated with the odds of asthma prevalence. The unadjusted model [0.96 (0.94, 0.97) *p* < 0.0001], the demographic-adjusted model [0.96 (0.94, 0.98) p < 0.0001], and the fully adjusted model [0.97 (0.95, 0.99) *p* = 0.0102] all indicated a negative association between PNI and asthma. Every unit rise in PNI was associated with a 3% reduction in asthma prevalence in the completely adjusted model (model 3). When PNI was classified as a quartile variable, the negative association remained. Compared to the baseline group, PNI quartiles 2 and 3 were associated with a 1% [0.99 (0.83, 1.18) *p* = 0.9180] and 14% [0.86 (0.71, 1.03) *p* = 0.1436] reduction in asthma prevalence, respectively, though these were not statistically significant (*p* > 0.05). Quartile 4 showed a significant 26% reduction [0.74 (0.59, 0.91) *p* = 0.0097] in asthma prevalence (*p* < 0.05), with a trend test *p*-value of 0.0041. Smooth fitting curves suggested a linear inverse association between PNI and asthma (Figure 2). Furthermore, we have delineated the existence of smooth fitting curves of PNI and asthma between different age groups and genders. As PNI increased, a tendency toward a decrease in asthma incidence was observed among subjects of various age groups (Figure 3). The relationship between PNI and asthma prevalence showed a similar overall non-linear trend in both males and females, characterized by a general decline in prevalence with increasing PNI values. However, the specific PNI values at which hotspots and subsequent declines occurred differed between genders. Notably, a rebound phenomenon was observed in males at higher PNI levels, where asthma prevalence increased again after an initial decline. This suggests potential gender-based disparities in how nutritional status impacts asthma manifestation (Figure 4). These findings imply that higher PNI levels may be indicative of a reduced risk of developing asthma.

**Table 2:** Associations between prognostic nutritional index and asthma.

Characteristic	Model 1 OR (95% CI) *N* = 7,869	Model 2 OR (95% CI) *N* = 7,869	Model 3 OR (95% CI) *N* = 7,869
PNI	0.96 (0.94, 0.97) <0.0001	0.96 (0.94, 0.98) <0.0001	0.97 (0.95, 0.99) 0.0102
Categories			
Q1	1.0	1.0	1.0
Q2	0.91 (0.77, 1.07) 0.2554	0.94 (0.80, 1.11) 0.4658	0.99 (0.83, 1.18) 0.9180
Q3	0.79 (0.66, 0.93) 0.0212	0.82 (0.69, 0.97) 0.0244	0.86 (0.71, 1.03) 0.1436
Q4	0.67 (0.57, 0.80) <0.0001	0.70 (0.58, 0.84) 0.0001	0.74 (0.59, 0.91) 0.0097
PNI group trend	<0.0001	<0.0001	0.0041

Model 1: no covariates were adjusted. Model 2: age, gender, and race were adjusted. Model 3: gender, age, race, family income-to-poverty ratio, education level, physical activity status, marital status, drink status, smoking status, systolic blood pressure, diastolic blood pressure, white blood cell count, Platelet count, cholesterol, triglycerides, HDL-Cholesterol, LDL-Cholesterol, body mass index, total calcium, high-sensitivity c-reactive protein, glycohemoglobin, fasting glucose, and history of diabetes, hypertension, and arthritis were adjusted. PNI, prognostic nutritional index; HDL-Cholesterol, high-density lipoprotein cholesterol; LDL-Cholesterol, low-density lipoprotein cholesterol.

### 3.3 Subgroup analysis

To evaluate the stability of the inverse correlation between PNI and asthma in the fully adjusted model, subgroup analyses by gender, age, race, BMI, diabetes, and hypertension were conducted. The results showed a negative association between PNI and asthma in all subgroups, with no significant interaction *p*-values, indicating the negative association was similar across different populations (Table 3).

**Table 3:** Subgroup analysis of the association between prognostic nutritional index and asthma.

Characteristic	Model OR (95% CI) *N* = 7,869	*p*-value	*p* for interaction
Stratified by age (years)			0.8197
20–40	0.98 (0.95, 1.01)	0.1513	
40–60	0.97 (0.93, 1.00)	0.0489	
60–80	0.98 (0.94, 1.01)	0.2404	
Stratified by gender			0.1958
Male	0.99 (0.95, 1.02)	0.3635	
Female	0.96 (0.94, 0.99)	0.0042	
Stratified by race			0.9115
Mexican American	0.99 (0.93, 1.04)	0.6225	
Other Hispanic	0.96 (0.91, 1.01)	0.1031	
Non-Hispanic White	0.98 (0.95, 1.01)	0.2070	
Non-Hispanic Black	0.96 (0.93, 1.00)	0.0500	
Other race	0.97 (0.92, 1.01)	0.1721	
Stratified by BMI			0.2860
Normal weight	0.99 (0.96, 1.02)	0.3372	
Overweight	0.96 (0.92, 0.99)	0.0175	
Obese	0.96 (0.92, 0.99)	0.0200	
Stratified by diabetes			0.5427
Yes	0.98 (0.94, 1.03)	0.4259	
No	0.97 (0.95, 0.99)	0.0089	
Stratified by high blood pressure			0.2652
Yes	0.98 (0.95, 1.01)	0.2488	
No	0.96 (0.94, 0.99)	0.0044	

Model: gender, age, race, family income-to-poverty ratio, education level, physical activity status, marital status, drink status, smoking status, systolic blood pressure, diastolic blood pressure, white blood cell count, Platelet count, cholesterol, triglycerides, HDL-Cholesterol, LDL-Cholesterol, body mass index, total calcium, high-sensitivity c-reactive protein, glycohemoglobin, fasting glucose, and history of diabetes, hypertension, and arthritis were adjusted. PNI, prognostic nutritional index; HDL-Cholesterol, high-density lipoprotein cholesterol; LDL-Cholesterol, low-density lipoprotein cholesterol.

## 4 Discussion

This study, including 7,869 adult participants from the United States, aimed to assess the association between PNI and asthma. We found that elevated PNI values were linked to a lower likelihood of asthma prevalence. The negative correlation between PNI and asthma remained consistent in the completely adjusted model. The results of the smooth curve analysis suggested a linear negative correlation between PNI and asthma. Subgroup analyses showed no significant interaction effects, suggesting that the negative association between PNI and asthma was consistent across different populations. Our study suggests that PNI can serve as an effective indicator for evaluating asthma prevalence. Early nutritional and inflammatory management in high-risk populations with low PNI levels may reduce the occurrence and severity of asthma.

PNI levels, which are evaluated through lymphocyte count and serum albumin, are associated with prognostic value in a variety of diseases (12, 13, 16, 17), but the relationship between PNI and asthma remains unclear. To the best of our knowledge, this is the inaugural cross-sectional study to examine the correlation between PNI and asthma. Asthma is a chronic inflammatory condition of the airways, characterized by the involvement of a diverse range of immune and inflammatory cells (18), with chronic inflammation playing a key role in asthma development (26). Since asthma symptoms cannot be completely cured, continuous long-term assessment of inflammatory response levels is crucial for controlling asthma occurrence and progression. T lymphocytes have been shown to play a crucial role in the immune response in asthma. Specifically, CD4+ T cells have been shown to drive the process of chronic inflammation and remodeling of the airways through the secretion of cytokines such as IL-4, IL-5, IL-13 and others (27), which not only promote the proliferation of the airway smooth muscle, but also may lead to structural changes. Different subpopulations of T lymphocytes may also play an important role in the immunophenotype and severity of asthma (28). T cell subset expressing archetypal granulocyte proteins, these subpopulations of T cells are uniquely characterized in the expression of granulocyte-associated proteins and may play an important role in chronic inflammation and remodeling of the airways. This suggests that changes in lymphocyte subpopulations may be closely related to the pathological progression of asthma and the state of immune function in patients. Furthermore, it has been determined that the airways of patients with allergic asthma are predominantly populated by TH2-type T-lymphocytes (29). The secretion of IL-4, IL-5 and IL-13 cytokines by these cells drives chronic inflammation and excessive immune responses in the airways, thereby further strengthening our understanding of TH2-type immune responses in asthma. Furthermore, PNI, as an indicator of immune status, may offer novel perspectives for evaluating the immune burden in asthma patients. A study indicated that the neutrophil-to-lymphocyte ratio (NLR) and platelet-to-lymphocyte ratio (PLR) are significant clinical indicators for diagnosing and monitoring bronchial asthma, with significantly lower lymphocyte counts and higher NLR and PLR levels in severe asthma groups compared to non-severe and healthy control groups (*p* < 0.0001) (30). Huang et al.’s meta-analysis also suggested that NLR is an effective inflammatory marker for predicting asthma and its acute exacerbations (31). Normal serum albumin levels have important antioxidant and anti-inflammatory effects (32). The selective inhibition of tumor necrosis factor-*α* (TNF-α) and its subsequent induction of vascular cell adhesion molecule expression by physiological levels of albumin has been demonstrated to reduce inflammatory processes (33). A correlation has been established between the severity of asthma and lower albumin levels. Furthermore, it is possible that this may also affect the unbound concentrations of certain drugs (34).

Nutritional status is closely related to inflammation levels, with malnutrition weakening immune system function (35), making it difficult for the body to effectively resist and regulate inflammation. A cohort study revealed a strong correlation between low serum albumin levels and an elevated risk of mortality in individuals with asthma (36). These findings were further supported by the observation that low albumin levels frequently coincided with immunocompromise and a chronic inflammatory state, which has been demonstrated to result in exacerbations and frequent acute exacerbations of asthma. A further study explored the relationship between protein intake, serum albumin levels and blood eosinophil counts in a group of asthmatic adults in the United States. The findings of this study indicated that low serum albumin levels were associated with increased eosinophil counts (37). Low albumin levels are typically indicative of chronic inflammation or malnutrition, and this condition may result in an imbalance in immune function, which in turn affects the clinical manifestations of asthma. A large-scale survey in Japan found that underweight asthma patients had poorer asthma control compared to those with normal weight (38). Malnutrition also increases oxidative stress levels in the body, which is a significant component of asthma inflammation (39). Oxidative stress and pro-inflammatory mediators play a key role in regulating gene expression by altering histone acetylation and deacetylation, affecting transcription factor binding to encyclopedia, and enhancing pro-inflammatory gene expression in various lung cells (40). Malnourished patients often lack specific nutrients. Some nutrients, such as vitamin D, vitamin C, and Omega-3 polyunsaturated fatty acids (PUFA), have been demonstrated to play crucial roles in regulating immune response and inflammation, improving lung function, and reducing wheezing (41). Asthma is often associated with enhanced type 2 T helper cell-mediated immune response. Vitamin D not only produces anti-inflammatory cytokines but also inhibits pro-inflammatory cytokines, thus maintaining equilibrium between type 1 T helper cells and type 2 T helper cells (42). Vitamin D can enhance the production of antimicrobial peptides in airway epithelial cells, reducing infection risk and asthma occurrence (43, 44). Vitamin C, as a potent antioxidant, can scavenge free radicals in the airways, reducing oxidative stress (45), and has anti-inflammatory effects independent of its antioxidant properties (46), helping to control inflammation in asthma patients, reduce airway inflammation, and mitigate asthma exacerbation severity (47, 48). Asthma patients have significantly lower levels of vitamin C in lung tissue and plasma compared to healthy controls, and lower vitamin C levels are associated with higher airway reactivity in asthma patients (49). Omega-3 PUFA, by converting into bioactive lipid molecules with anti-inflammatory effects, such as prostaglandins, leukotrienes, and resolvins, reduce the production of inflammatory cytokines and type 2 t helper cell activity, reducing airway inflammation and hyperresponsiveness (50, 51). Even if the mother has asthma, maternal intake of oily fish during pregnancy can reduce the risk of asthma in children (52, 53). Studies have shown that dietary nutrition improvement can significantly reduce inflammation levels in asthma patients (54). Obesity represents a multifaceted risk factor for asthma, exerting a potential influence on asthma severity and control through a number of mechanisms. For instance, obesity may result in chronic low-grade inflammation, heightened airway inflammation and reactivity, which in turn may influence asthma symptoms and treatment outcomes. The distinctive phenotype of obesity-asthma, particularly in certain obese asthmatic individuals, is frequently associated with diminished control, suboptimal response to medications, and additional characteristics (55). Asthmatic patients exhibit considerably lower hemoglobin concentrations compared to non-asthmatic individuals (56). These factors, including malnutrition, low serum albumin, overweight, and anemia, may contribute to exacerbated or poorly controlled asthma symptoms through mechanisms such as exacerbation of chronic inflammation and immune response. The impact of nutritional status on asthma is multifaceted, including immune function, inflammatory response, oxidative stress, and specific nutrient effects. Low PNI reflects poor nutritional and immune status, potentially leading to more severe asthma symptoms and faster disease progression.

The present study revealed a decline in both male and female subjects, although the specific PNI values at which these declines occurred differed by gender. At lower PNI values, the prevalence of asthma was higher in males, and as PNI values increased, there was a rebound in asthma prevalence in males and an overall decreasing trend in females. This finding suggests the potential for a sex-based disparity in the relationship between PNI and asthma. This gender disparity may be attributed to the differential mechanisms of action of sex hormones, with fluctuations in estrogen and progesterone levels playing a pivotal role in asthma susceptibility and clinical manifestations in women (57). Estrogens have been demonstrated to elicit a divergent response to asthma in women compared to men, with the potential to influence immune system function. In experimental studies utilizing murine models, Estrogens have been observed to contribute to asthma by facilitating Th2-mediated immune responses, increasing eosinophil recruitment, and enhancing asthma prevalence in women by promoting Th2-mediated immune responses, increasing eosinophil recruitment, and enhancing asthma prevalence in women. Acidophilic granulocyte recruitment and enhanced cytokine production exacerbating asthma-like inflammation (58). However, sexual maturation has been reported to prevent lung inflammation under specific experimental conditions, this finding underscores the dual role of estrogen, which may be contingent on the specific estrogen, the environmental context, and the diurnal phase (59). Moreover, elevated levels of PNI can trigger heightened androgen levels, which, in turn, may lead to overactivation of ERK signaling. This, in turn, can promote leukotriene synthesis under certain conditions (60). The aforementioned factors may provide a rationale for the observed gender disparities in the prevalence of PNI and asthma. This finding underscores the necessity of incorporating nutritional and hormonal factors into comprehensive asthma management strategies.

The principal strengths of our study are the large sample size and the representativeness of the data, making the results widely applicable. In order to enhance the validity and applicability of the findings, numerous confounding variables were also taken into consideration. However, limitations include the definition of asthma based on questionnaires, which cannot accurately classify asthma phenotypes, and the cross-sectional design, which does not establish causality between PNI and asthma. Furthermore, it is acknowledged that the NHANES data does not provide a classification of asthma severity (i.e., mild, moderate or severe) or details on the degree of asthma control. As asthma severity is often associated with nutritional status, particularly in more severe cases where malnutrition is more likely, this lack of severity data may indeed lead to bias in the findings, which could confound the relationship between PNI and asthma outcomes.

## 5 Conclusion

Our study indicates that lower PNI levels are associated with an increase in the odds of prevalence of asthma. Early nutritional guidance for high-risk populations can effectively improve nutritional status, regulate antioxidant and immune capabilities, and reduce the occurrence of asthma. Future prospective studies are needed to validate this relationship.

## Data Availability

Publicly available datasets were analyzed in this study. This data can be found here: www.cdc.gov/nchs/nhanes/.

## References

[1] Pelaia C Zannoni E Paoletti G Marzio V Heffler E Carrón-Herrero A. Clinical remission in severe asthma: lights and shadows on an ambitious goal. Curr Opin Allergy Clin Immunol. (2024) 24:230–6. doi: 10.1097/ACI.0000000000000991, PMID: 38713864

[2] GBD 2019 Chronic Respiratory Diseases Collaborators. Global burden of chronic respiratory diseases and risk factors, 1990-2019: an update from the global burden of disease study 2019. EClinicalMedicine. (2023) 59:101936. doi: 10.1016/j.eclinm.2023.101936, PMID: 37229504 PMC7614570

[3] Pate CA Zahran HS Qin X Johnson C Hummelman E Malilay J. Asthma surveillance - United States, 2006-2018. MMWR Surveill Summ. (2021) 70:1–32. doi: 10.15585/mmwr.ss7005a1, PMID: 34529643 PMC8480992

[4] Covantev S Mazuruc N Uzdenov R Corlateanu A. Spontaneous Pneumomediastinum – a rare asthma complication. Folia Med. (2019) 61:472–7. doi: 10.3897/folmed.61.e39419, PMID: 32337937

[5] Yang G Han YY Forno E Yan Q Rosser F Chen W. Glycated hemoglobin a(1c), lung function, and hospitalizations among adults with asthma. J Allergy Clin Immunol Pract. (2020) 8:3409–3415.e1. doi: 10.1016/j.jaip.2020.06.017, PMID: 32569755 PMC7655696

[6] Raita Y Camargo CA Jr Faridi MK Brown DFM Shimada YJ Hasegawa K. Risk of acute myocardial infarction and ischemic stroke in patients with Asthma exacerbation: a population-based, self-controlled case series study. J Allergy Clin Immunol Pract. (2020) 8:188–194.e8. doi: 10.1016/j.jaip.2019.06.043, PMID: 31323338

[7] van Meel ER Jaddoe VWV Bønnelykke K de Jongste JC Duijts L. The role of respiratory tract infections and the microbiome in the development of asthma: a narrative review. Pediatr Pulmonol. (2017) 52:1363–70. doi: 10.1002/ppul.23795, PMID: 28869358 PMC7168085

[8] Yang CH Lv JJ Li XY Yang XT Yin MY. Global burden of asthma in young adults in 204 countries and territories, 1990-2019: systematic analysis of the global burden of disease study 2019. Prev Med Rep. (2024) 37:102531. doi: 10.1016/j.pmedr.2023.102531, PMID: 38162120 PMC10755496

[9] Ali Z Ulrik CS. Obesity and asthma: a coincidence or a causal relationship? A systematic review. Respir Med. (2013) 107:1287–300. doi: 10.1016/j.rmed.2013.03.019 23642708

[10] Choi HG Kim JH Park JY Hwang YI Jang SH Jung KS. Association between asthma and depression: a national cohort study. J Allergy Clin Immunol Pract. (2019) 7:1239–1245.e1. doi: 10.1016/j.jaip.2018.10.046 30423450

[11] Lomper K Chudiak A Uchmanowicz I Rosińczuk J Jankowska-Polanska B. Effects of depression and anxiety on asthma-related quality of life. Pneumonol Alergol Pol. (2016) 84:212–21. doi: 10.5603/PiAP.2016.0026, PMID: 27435347

[12] Cao D Dong Q. Predictive value of prognostic nutritional index for outcomes of cervical cancer: a systematic review and meta-analysis. Exp Ther Med. (2024) 28:316. doi: 10.3892/etm.2024.12605, PMID: 38939175 PMC11209845

[13] Sun CY Zhang XJ Li Z Fei H Li ZF Zhao DB. Preoperative prognostic nutritional index predicts long-term outcomes of patients with ampullary adenocarcinoma after curative pancreatoduodenectomy. World J Gastrointest Surg. (2024) 16:1291–300. doi: 10.4240/wjgs.v16.i5.1291, PMID: 38817277 PMC11135320

[14] Keskinkilic M Semiz HS Ataca E Yavuzsen T. The prognostic value of immune-nutritional status in metastatic colorectal cancer: prognostic nutritional index (PNI). Support Care Cancer. (2024) 32:374. doi: 10.1007/s00520-024-08572-6, PMID: 38777931 PMC11111560

[15] Ma H Liu Y Ye H Gao F Li Z Qin S. The prognostic value of preoperative laboratory data indicators in patients with esophageal carcinoma: an observational study. Medicine. (2024) 103:e38477. doi: 10.1097/MD.0000000000038477, PMID: 38875403 PMC11175890

[16] Zhou J Ma L Zhao L Sheng J Xu Y Chen J. Association between the prognostic nutritional index and cognitive function among older adults in the United States: a population-based study. J Alzheimers Dis. (2021) 83:819–31. doi: 10.3233/JAD-210141, PMID: 34366335

[17] Jhang SW Liu YT Kor CT Wu YP Lai CH. Low prognostic nutritional index predicts in-hospital complications and case fatality in patients with spontaneous intracerebral hemorrhage: a retrospective study. Nutrients. (2024) 16:1841. doi: 10.3390/nu16121841, PMID: 38931196 PMC11206377

[18] Lee YF Lin PR Wu SH Hsu HH Yang SY Kor CT. Impact of the prognostic nutritional index on renal replacement therapy-free survival and mortality in patients on continuous renal replacement therapy. Ren Fail. (2024) 46:2365394. doi: 10.1080/0886022X.2024.2365394, PMID: 38874108 PMC11232640

[19] Wu TT Pan Y Zhi XY Deng CJ Wang S Guo XX. Association between extremely high prognostic nutritional index and all-cause mortality in patients with coronary artery disease: secondary analysis of a prospective cohort study in China. BMJ Open. (2024) 14:e079954. doi: 10.1136/bmjopen-2023-079954, PMID: 38885991 PMC11184201

[20] Bousquet J Jeffery PK Busse WW Johnson M Vignola AM. From bronchoconstriction to airways inflammation and remodeling. Am J Respir Crit Care Med. (2000) 161:1720–45. doi: 10.1164/ajrccm.161.5.9903102, PMID: 10806180

[21] Alwarith J Kahleova H Crosby L Brooks A Brandon L Levin SM. The role of nutrition in asthma prevention and treatment. Nutr Rev. (2020) 78:928–38. doi: 10.1093/nutrit/nuaa005, PMID: 32167552 PMC7550896

[22] Veldhorst M Smeets A Soenen S Hochstenbach-Waelen A Hursel R Diepvens K. Protein-induced satiety: effects and mechanisms of different proteins. Physiol Behav. (2008) 94:300–7. doi: 10.1016/j.physbeh.2008.01.003, PMID: 18282589

[23] Nurmatov U Devereux G Sheikh A. Nutrients and foods for the primary prevention of asthma and allergy: systematic review and meta-analysis. J Allergy Clin Immunol. (2011) 127:724–733.e30. doi: 10.1016/j.jaci.2010.11.001, PMID: 21185068

[24] Varraso R Fung TT Barr RG Hu FB Willett W Camargo CA Jr. Prospective study of dietary patterns and chronic obstructive pulmonary disease among US women. Am J Clin Nutr. (2007) 86:488–95. doi: 10.1093/ajcn/86.2.488, PMID: 17684223 PMC2643338

[25] Onodera T Goseki N Kosaki G. Prognostic nutritional index in gastrointestinal surgery of malnourished cancer patients. Nihon Geka Gakkai Zasshi. (1984) 85:1001–5. PMID: 6438478

[26] Banno A Reddy AT Lakshmi SP Reddy RC. Bidirectional interaction of airway epithelial remodeling and inflammation in asthma. Clin Sci. (2020) 134:1063–79. doi: 10.1042/CS20191309, PMID: 32369100

[27] Amin K. The role of the T lymphocytes and remodeling in Asthma. Inflammation. (2016) 39:1475–82. doi: 10.1007/s10753-016-0380-9, PMID: 27221139

[28] Vázquez-Mera S Martelo-Vidal L Miguéns-Suárez P Bravo SB Saavedra-Nieves P Arias P. Exploring CD26(−/lo) subpopulations of lymphocytes in asthma phenotype and severity: a novel CD4(+) T cell subset expressing archetypical granulocyte proteins. Allergy. (2024) 79:3005–21. doi: 10.1111/all.16327, PMID: 39319599

[29] Robinson DS Hamid Q Ying S Tsicopoulos A Barkans J Bentley AM. Predominant TH2-like bronchoalveolar T-lymphocyte population in atopic asthma. N Engl J Med. (1992) 326:298–304. doi: 10.1056/NEJM199201303260504 1530827

[30] Shi G Zhao JW Ming L. Clinical significance of peripheral blood neutrophil-lymphocyte ratio and platelet-lymphocyte ratio in patients with asthma. Nan Fang Yi Ke Da Xue Xue Bao. (2017) 37:84–8. doi: 10.3969/j.issn.1673-4254.2017.01.15 PMID: 28109104 PMC6765764

[31] Huang WJ Huang GT Zhan QM Chen JL Luo WT Wu LH. The neutrophil to lymphocyte ratio as a novel predictor of asthma and its exacerbation: a systematic review and meta-analysis. Eur Rev Med Pharmacol Sci. (2020) 24:11719–28. doi: 10.26355/eurrev_202011_23819, PMID: 33275241

[32] Arques S. Human serum albumin in cardiovascular diseases. Eur J Intern Med. (2018) 52:8–12. doi: 10.1016/j.ejim.2018.04.014, PMID: 29680174

[33] Zhang WJ Frei B. Albumin selectively inhibits TNF alpha-induced expression of vascular cell adhesion molecule-1 in human aortic endothelial cells. Cardiovasc Res. (2002) 55:820–9. doi: 10.1016/S0008-6363(02)00492-3, PMID: 12176131

[34] Picado C Deulofeu R Lleonart R Agustí M Casals E Quintó L. Lipid and protein metabolism in asthma. Effects of diet and corticosteroid therapy. Allergy. (1999) 54:569–75. doi: 10.1034/j.1398-9995.1999.00024.x, PMID: 10435470

[35] Martínez de Toda I Ceprián N Díaz-Del Cerro E De la Fuente M. The role of immune cells in Oxi-Inflamm-aging. Cells. (2021) 10:2974. doi: 10.3390/cells10112974, PMID: 34831197 PMC8616159

[36] Zhuang R Liao J Giri M Wen J Guo S. Relationship between dietary protein, serum albumin, and mortality in asthmatic populations: a cohort study. Front Immunol. (2024) 15:1396740. doi: 10.3389/fimmu.2024.1396740, PMID: 39026682 PMC11254659

[37] Wen J Xia J He Q Giri M Guo S. Association between protein intake, serum albumin and blood eosinophil in US asthmatic adults. Front Immunol. (2024) 15:1383122. doi: 10.3389/fimmu.2024.1383122, PMID: 38835754 PMC11148351

[38] Furukawa T Hasegawa T Suzuki K Koya T Sakagami T Youkou A. Influence of underweight on asthma control. Allergol Int. (2012) 61:489–96. doi: 10.2332/allergolint.12-OA-0425, PMID: 22824977

[39] Wood LG Gibson PG Garg ML. Biomarkers of lipid peroxidation, airway inflammation and asthma. Eur Respir J. (2003) 21:177–86. doi: 10.1183/09031936.03.00017003a, PMID: 12570126

[40] Rahman I. Oxidative stress, chromatin remodeling and gene transcription in inflammation and chronic lung diseases. J Biochem Mol Biol. (2003) 36:95–109. doi: 10.5483/bmbrep.2003.36.1.095 PMID: 12542980

[41] Berthon BS Wood LG. Nutrition and respiratory health—feature review. Nutrients. (2015) 7:1618–43. doi: 10.3390/nu7031618, PMID: 25751820 PMC4377870

[42] Pichler J Gerstmayr M Szépfalusi Z Urbanek R Peterlik M Willheim M. 1 alpha, 25(OH)2D3 inhibits not only Th1 but also Th2 differentiation in human cord blood T cells. Pediatr Res. (2002) 52:12–8. doi: 10.1203/00006450-200207000-00005 PMID: 12084841

[43] Foong RE Zosky GR. Vitamin D deficiency and the lung: disease initiator or disease modifier? Nutrients. (2013) 5:2880–900. doi: 10.3390/nu5082880, PMID: 23896653 PMC3775233

[44] Hiemstra PS. The role of epithelial beta-defensins and cathelicidins in host defense of the lung. Exp Lung Res. (2007) 33:537–42. doi: 10.1080/01902140701756687, PMID: 18075828

[45] Hatch GE. Asthma, inhaled oxidants, and dietary antioxidants. Am J Clin Nutr. (1995) 61:625s–30s. doi: 10.1093/ajcn/61.3.625S 7879729

[46] Bowie AG O’Neill LA. Vitamin C inhibits NF-kappa B activation by TNF via the activation of p38 mitogen-activated protein kinase. J Immunol. (2000) 165:7180–8. doi: 10.4049/jimmunol.165.12.7180, PMID: 11120850

[47] Chang HH Chen CS Lin JY. High dose vitamin C supplementation increases the Th1/Th2 cytokine secretion ratio, but decreases eosinophilic infiltration in bronchoalveolar lavage fluid of ovalbumin-sensitized and challenged mice. J Agric Food Chem. (2009) 57:10471–6. doi: 10.1021/jf902403p, PMID: 19831405

[48] Jeong YJ Kim JH Kang JS Lee WJ Hwang YI. Mega-dose vitamin C attenuated lung inflammation in mouse asthma model. Anat Cell Biol. (2010) 43:294–302. doi: 10.5115/acb.2010.43.4.294, PMID: 21267403 PMC3026181

[49] Kelly FJ Mudway I Blomberg A Frew A Sandström T. Altered lung antioxidant status in patients with mild asthma. Lancet. (1999) 354:482–3. doi: 10.1016/S0140-6736(99)01812-7 10465176

[50] Kelley DS Taylor PC Nelson GJ Schmidt PC Ferretti A Erickson KL. Docosahexaenoic acid ingestion inhibits natural killer cell activity and production of inflammatory mediators in young healthy men. Lipids. (1999) 34:317–24. doi: 10.1007/s11745-999-0369-5, PMID: 10443964

[51] Calder PC. N-3 polyunsaturated fatty acids, inflammation, and inflammatory diseases. Am J Clin Nutr. (2006) 83:1505s–19s. doi: 10.1093/ajcn/83.6.1505S, PMID: 16841861

[52] Salam MT Li YF Langholz B Gilliland FD. Maternal fish consumption during pregnancy and risk of early childhood asthma. J Asthma. (2005) 42:513–8. doi: 10.1081/JAS-200067619 16293548

[53] Klemens CM Berman DR Mozurkewich EL. The effect of perinatal omega-3 fatty acid supplementation on inflammatory markers and allergic diseases: a systematic review. BJOG. (2011) 118:916–25. doi: 10.1111/j.1471-0528.2010.02846.x 21658192

[54] Nygaard UC Xiao L Nadeau KC Hew KM Lv N Camargo CA. Improved diet quality is associated with decreased concentrations of inflammatory markers in adults with uncontrolled asthma. Am J Clin Nutr. (2021) 114:1012–27. doi: 10.1093/ajcn/nqab063, PMID: 33871602 PMC8578836

[55] Granell R Henderson AJ Evans DM Smith GD Ness AR Lewis S. Effects of BMI, fat mass, and lean mass on asthma in childhood: a Mendelian randomization study. PLoS Med. (2014) 11:e1001669. doi: 10.1371/journal.pmed.1001669, PMID: 24983943 PMC4077660

[56] Nasreen S Nessa A Islam MF Husain MF Khatun N Wahed F. Relationship of hemoglobin concentration in adult asthmatic patients. Mymensingh Med J. (2016) 25:601–6. PMID: 27941716

[57] Baldaçara RP Silva I. Association between asthma and female sex hormones. Sao Paulo Med J. (2017) 135:4–14. doi: 10.1590/1516-3180.2016.011827016, PMID: 28076614 PMC9969728

[58] El-Desouki NI Tabl GA Elkhodary YA. Biological studies on the effect of estrogen on experimentally induced asthma in mice. Toxicol Ind Health. (2016) 32:30–8. doi: 10.1177/0748233713486959, PMID: 23863957

[59] Draijer C Hylkema MN Boorsma CE Klok PA Robbe P Timens W. Sexual maturation protects against development of lung inflammation through estrogen. Am J Physiol Lung Cell Mol Physiol. (2016) 310:L166–74. doi: 10.1152/ajplung.00119.2015, PMID: 26608529

[60] Pergola C Dodt G Rossi A Neunhoeffer E Lawrenz B Northoff H. ERK-mediated regulation of leukotriene biosynthesis by androgens: a molecular basis for gender differences in inflammation and asthma. Proc Natl Acad Sci USA. (2008) 105:19881–6. doi: 10.1073/pnas.0809120105, PMID: 19064924 PMC2597692

